# Hook Breathing Facilitates SaO_2_ Recovery After Deep Dives in Freedivers With Slow Recovery

**DOI:** 10.3389/fphys.2019.01076

**Published:** 2019-08-30

**Authors:** Fran de Asís Fernández, Lara Rodríguez-Zamora, Erika Schagatay

**Affiliations:** ^1^Departament of Health, Centro Superior de Estudios Universitarios La Salle, Universidad Autónoma de Madrid, Madrid, Spain; ^2^Department of Health Sciences, Mid Sweden University, Östersund, Sweden; ^3^Department of Health and Medical Sciences, Division of Sport Sciences, Örebro University, Örebro, Sweden

**Keywords:** apnea, breath-hold, respiration, hypoxia, syncope, blackout, pulmonary edema, freediving safety

## Abstract

To facilitate recovery from hypoxia, many freedivers use a breathing method called “hook breathing” (HB) after diving, involving an interrupted exhale to build up intrapulmonary pressure. Some divers experience a delay in recovery of arterial oxygen saturation (SaO_2_) after diving, interpreted as symptoms of mild pulmonary edema, and facilitated recovery may be especially important in this group to avoid hypoxic “blackout.” We examined the influence of HB on recovery of SaO_2_ in freedivers with slow recovery (SR) and fast recovery (FR) of SaO_2_ after deep “free immersion” (FIM) apnea dives to 30 m depth. Twenty-two male freedivers, with a mean (SD) personal best in the discipline FIM of 57(26) m, performed two 30 m deep dives, one followed by HB and one using normal breathing (NB) during recovery, at different days and weighted order. SaO_2_ and heart rate (HR) were measured *via* pulse oximetry during recovery. The SR group (*n* = 5) had a faster SaO_2_ recovery using HB, while the FR group (*n* = 17) showed no difference between breathing techniques. At 105 s, the SR group reached a mean (SD) SaO_2_ of 95(5)% using HB, while using NB, their SaO_2_ was 87(5)% (*p* < 0.05), and 105–120 s after surfacing SaO_2_ was higher with HB (*p* < 0.05). In SR subjects, the average time needed to reach 95% SaO_2_ with HB was 60 s, while it was 120 s at NB (*p* < 0.05). HR was similar in the SR group, while it was initially elevated at HB in the FR group (*p* < 0.05). We conclude that HB efficiently increases SaO_2_ recovery in SR individuals, but not in the FR group. The proposed mechanism is that increased pulmonary pressure with HB will reverse any pulmonary edema and facilitate oxygen uptake in divers with delayed recovery.

## Introduction

Breath-hold diving, also called “apnea diving” or “freediving” involves diving without a breathing apparatus or other external air supply, and is not only used professionally to obtain food, but increasingly popular as a sports activity, both for recreation and competition. During freediving, the O_2_ reserves are progressively reduced and during ascent from depth arterial oxygen saturation (SaO_2_) falls rapidly, with high risk of loss of consciousness (Albert and Craig, 1961) often called blackout (BO), near the end of maximal dives, or after surfacing. Without assistance, this may lead to drowning. In competitive events, this risk is nearly eliminated by the use of safety divers (Fitz-Clarke, 2006), but pulmonary edema may compromise recovery and has led to one lethal outcome in competition. A major concern among divers is to surface with a margin to BO, as this risk may remain up to 1 min due to the circulatory delay and inefficient recovery. Incidents due to hypoxia may thus occur after the diver has surfaced (Lindholm, 2007) and BO in apnea competitions also after surfacing leads to disqualification. A “surface protocol” has to be presented by the divers on surfacing, requiring that the diver is not too hypoxic, and divers failing to complete it correctly are also disqualified. This protocol has been introduced by AIDA international (the main apnea competition organizer) to make the divers more conservative with announcing depths.

Aiming to reduce the risk of BO, many apnea divers use a specialized breathing technique after surfacing called “Hook Breathing” (HB; Schagatay, 2011). The technique involves a deep inspiration with the breathing cycle interrupted early during exhale with a maneuver similar to Valsalva (Lee et al., 1954) and with the subsequent expiration performed against resistance in order to generate continuous positive airway pressure during exhalation. Similar breathing patterns after diving have been observed in two groups of professional breath-hold divers in Asia, the Ama of Japan (Rahn and Yokoyama, 1965) and the Sama-Bajau divers in Indonesia (Schagatay, unpublished observations 2017). HB is also used among Air Force fighter pilots in order to avoid the loss of consciousness at elevated G-forces due to drop in blood pressure during acceleration (Whinnery and Murray, 1990).

In addition to BO, a problem linked with deep apnea diving is the risk to develop pulmonary edema, and rupture of blood vessels leading to bleeding in lungs or airways (Boussuges et al., 1999; Lindholm et al., 2008). These conditions are collectively called “squeeze” by the divers, despite their somewhat different manifestations (Kalemoglu and Keskin, 2006). Lung squeeze may contribute to the risk of BO, by compromising gas exchange. The cause of lung squeeze in deep freediving is mainly related to the development of lower pressure in the lungs than in the surrounding capillaries due to the reduced air volume as an effect of the increasing hydrostatic pressure with depth (Schaefer et al., 1968). Until the lung volume has been reduced to residual volume, the intrathoracic pressure is equal to hydrostatic pressure, but below this depth, lung volume can no longer shrink due to the chest rigidity, and the decreasing intrathoracic pressure then causes a “blood shift” to the thoracic vessels in proportion to the depth, and further increasing depth will cause an increase of the transpulmonary capillary wall pressure leading to pulmonary edema or hemorrhage (Boussuges et al., 1999).

These responses to depth are highly individual, with 80% of the squeeze injuries occurring in 20% of the divers (Cialoni et al., 2012). While the cause of these individual differences is unknown, risk thus becomes evident when lungs are compressed below RV, which may occur at 25–40 m depending on the diver’s lung volumes and mechanics (Schaefer et al., 1968). The progressively increasing depths reached in competition have made the occurrences of squeeze more common (Lindholm et al., 2008). It has been found that SaO_2_ during recovery gives an indication on which divers suffer from squeeze (Linér and Andersson, 2008; Schagatay et al., 2015), and AIDA international has, based on these findings, incorporated systematic recording of SaO_2_ after deep competition dives (AIDA-International, 2017). Normal SaO_2_ should be above 95% (Wood, 1949), and since 2015, the responsible medical doctor may in consultation with the judge restrict continued diving in divers not having reached 95% SaO_2_ within 20 min after surfacing as lower values have been found to be associated with symptoms of lung squeeze (Schagatay et al., 2015).

In freedivers, surfacing with low SaO_2_, the first breaths must provide fast and efficient restitution of the normal oxygen levels to prevent BO. Some divers experience a delay in recovery of SaO_2_ after diving, and facilitated recovery may be especially important in this group. Several mechanisms increasing SaO_2_ could be associated with HB. The continuous positive airway pressure during exhalation could open up atelectatic areas of the lung and force out the fluid associated with edema, and the increased partial pressure of O_2_ achieved in the alveoli with HB would likely increase gas exchange. During exhale after diving, blood pressure falls, which could also contribute to BO, and likely be counteracted by HB. However, this common breathing technique among freedivers awaits scientific analysis.

The purpose of this study was therefore to examine the influence of HB on SaO_2_ recovery after deep diving compared to normal breathing (NB), in divers with slow recovery (SR) and fast recovery (FR). We hypothesize that there will be faster SaO_2_ recovery associated with HB, and that these beneficial effects will be more pronounce in divers with delayed recovery.

## Materials and Methods

### Methods Development

While delayed SaO_2_ recovery after diving has previously been connected to various symptoms of lung barotrauma (Schagatay et al., 2015), its reliability to detect pulmonary edema has not previously been systematically evaluated using established clinical methods. In order to assess pulse oximetry for identifying subjects with delayed recovery due to mild pulmonary edema, we did a pilot study using SaO_2_ and lung ultrasound in parallel. With lung ultrasound, lung “comets” can be counted on images of different lung sections to quantify the occurrence of lung water, reflecting pulmonary edema (Frassi et al., 2008). Nine-freedivers did 1–2 near maximal or maximal deep dives each, as part of their training before a competition. Diving depth varied from 58 to 88 m, with a mean (SD) of 77(8) m. Diving disciplines included were “constant weight” (CWT; 4 dives) where the diver swims down along a vertical line to a pre-set target depth and up again using a monofin, “variable weight” (VW; 3 dives) where the freediver uses a weighted sled on the way down but swims up using a monofin, and “free immersion” (FIM; 2 dives) where the diver pulls him/herself down and up along a vertical line using only the arms. At a total of 35 time points from 2 to 60 min after diver’s surfacing, we measured SaO_2_ with pulse oximetry and simultaneously used lung ultrasound for comet calculations. Divers presented with 0–154 comets, and SaO_2_ varied between 98.2 and 91.4%. There was a strong negative correlation between post dive lung comets and SaO_2_ (*r* = −0.67; *p* < 0.001). We concluded that low SaO_2_ after diving indicates pulmonary edema, and that SaO_2_ can be used as a field method to determine the level of edema in a freediver.

### Study Design

There are several pulmonary symptoms related to barotrauma in freedivers, including delayed SaO_2_recovery. Cialoni et al. (2012) found that all divers reporting such symptoms reported several events through their diving history, and that incidence correlated to their personal best dive registered in an official deep apnea championship. The incidence of symptoms in divers with a personal best of 36–45 m was 47%, in divers with a personal best of 46–55 m it was 65% and for 56–76 m it was nearly 80% (Cialoni et al., 2012). Aiming to identify a sufficient number of divers with delayed SaO_2_ recovery, we wanted to include 20–25 male freedivers, with a personal best in FIM of over 45 m in the study. The sample size of the study has been determined according to *α* level, *p* = 0.05, power 80%.

The depth used in the experimental dives should be close enough to the diver’s personal best to have a possible effect on the lungs, but with a safety margin to avoid serious barotrauma. We also wanted the divers to be able to repeat the same depth on 2 days without warm up dives, and 30 m was considered to be easy enough to achieve this.

The reasons for using dives in the discipline FIM were two; first that the discipline has been shown to be associated with a greater incidence of lung symptoms (Schagatay et al., 2009), and second, that the pulling down and up on a line characteristic of this discipline gives best control of both depth and speed compared to other disciplines. The two dives were separated by 24 h and the starting order of the breathing maneuvers (HB or NB) was weighted between subjects, to avoid any order effects or tiredness of the divers.

### Participants

Twenty-two male breath-hold divers volunteered during a training week before an international freediving competition. Their mean (SD) age, weight, and height were 34(7) years, 76(7) kg and 178(7) cm, respectively. The divers mean (SD) apnea experience in competitive apnea was 6(4) years in FR and 8(4) years in SR; on the other hand, the personal best in FIM was 56(25) m in FR and 59(20) m in SR. Participants resting SaO_2_ was determined before dives, and two subjects with a pre-dive SaO_2_ below 95% were excluded from further study. The mean (SD) SaO_2_ values during baseline was 96.2(1)% in FR and 95.4(1)% in SR.

Divers were informed of the procedures including benefits and risks prior to signing the informed consent document. The study was conducted in accordance with the Declaration of Helsinki (Harriss and Atkinson, 2011) and was approved by the local Human Research Ethics Committee (CEI-70-1257).

### Study Procedures

**Figure 1:**
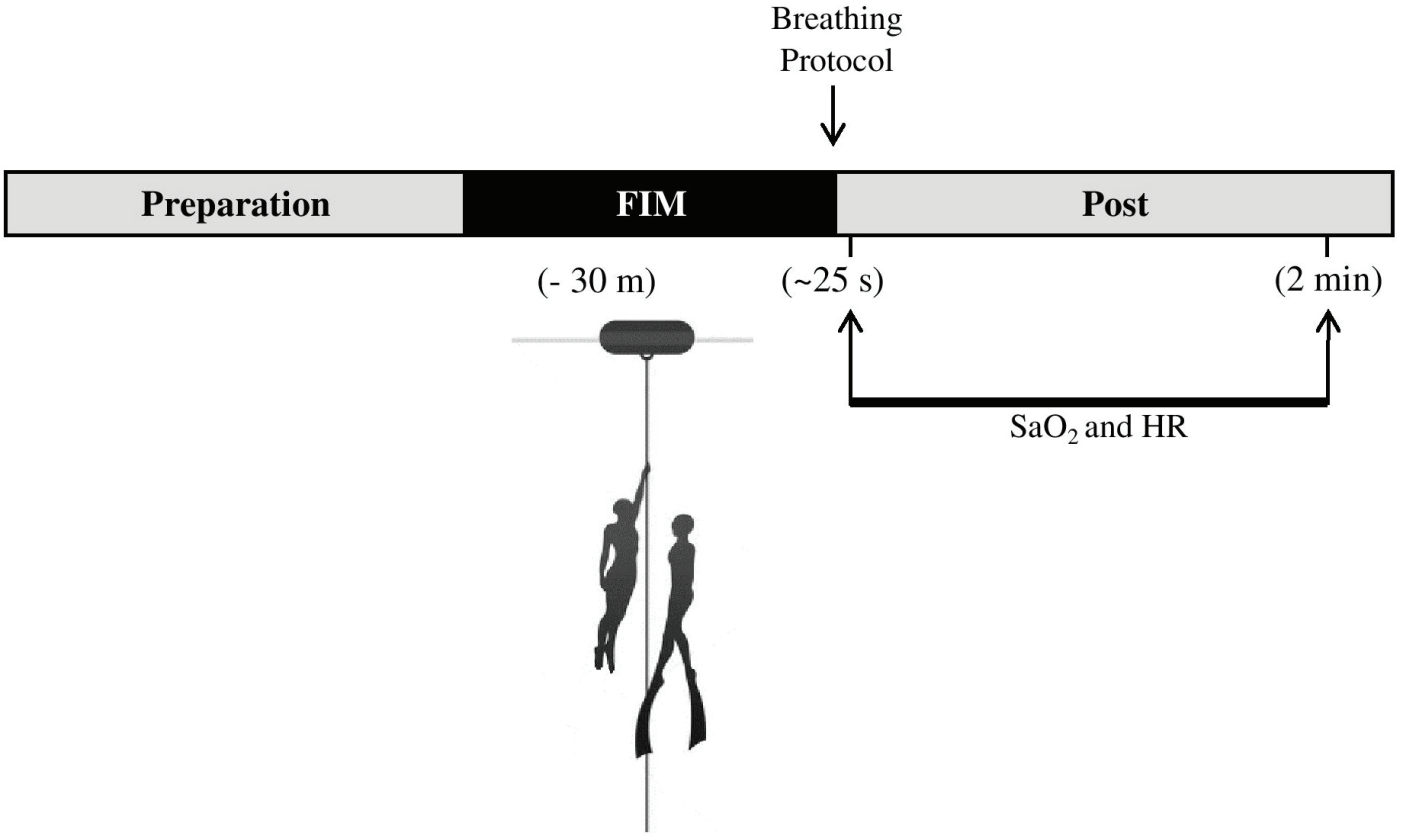
Study protocol for monitoring arterial oxygen saturation (SaO_2_) and heart rate (HR) after 30 m dives by free immersion (FIM) followed by 2 min recovery breathing using either hook breathing (HB) or normal breathing (NB).

Each freediver performed two FIM dives to 30 m depth at different days, one followed by recovery breathing using HB and the other using NB (described below). The starting order of the breathing maneuvers (HB or NB) was weighted, so that every other diver started with HB. Previous to the immersion, while divers rested on the dive buoy close to the research boat, they were informed of which breathing protocol to use (HB or NB) during recovery. Divers used their normal breathing preparation at the surface before dives, but without overfilling the lungs by “lung packing” (Örnhagen et al., 1998). Divers then used the arms to pull themselves down along the vertical line and up again followed by recovery breathing using either HB or NB (Figure 1). Dives were done at a controlled slow pace, with a duration of around 2 min per dive. The diver was asked to start the dive when feeling ready.

**Figure 2:**
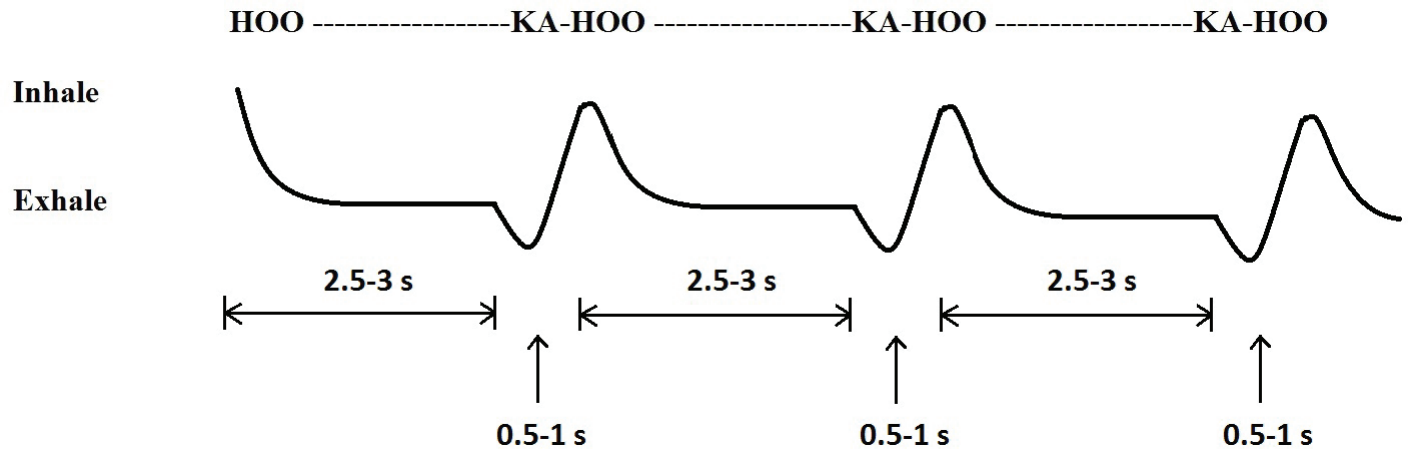
The timing of the respiratory components during performance of hook breathing. This technique involves a deep inspiration with the breathing cycle interrupted early during exhale and with the subsequent expiration performed against resistance.

Most divers were already familiar with the HB protocol but they all received the following information: Inhale and begin to say the word “Hook.” Before hitting the “k” sound, close your glottis and hold your breath. Exhale, finish the word “hook,” and make the “k” come out like “k-ah” (Figure 2). Then inhale and repeat. Continue the technique until you feel recovered, but for at least 2 min. During NB, divers performed breaths as normal without interrupting the exhalations.

A safety diver was accompanying the test divers during ascent, as done during competitions. A bottom plate was set at 30 m, with a lanyard attaching the diver to the line for safety. Divers surfaced on the buoy then moved a few meters to the side of the research boat where SaO_2_and HR recordings started as soon as practically possible after surfacing. HR and SaO_2_ were recorded by the experimenters every 5 s during 2 min by a handheld pulse oximeter (NoninPalmSAT^®^ 2500 series, Nonin Medical Inc.) while divers performed either the HB or NB protocol. During the measurement, a signal on the pulse oximeter indicated with green light or red light if readings were reliable. At the start of measurement, there was sometimes a poor signal before measurement stabilized, possibly due to a remaining diving-related vasoconstriction. Only data with good readings indicated were included.

### Statistical Analysis

The SaO_2_ recordings started at different time points for different individuals, but data from all subjects were complete from 25 s post dive, and analysis is therefore based on values for 25–120 s. Five of the subjects were identified as SR, by being below 95% after 2 min in one or both dives, and the groups analyzed separately henceforth (Supplementary Table S1). Personal best in FIM was 59(20) in the SR group and 56(25) in the FR group (NS). Results were expressed as means with standard deviation (SD). Normality was assessed using the Shapiro-Wilk’s test. A two-way mixed ANOVA was used to evaluate the effect of the breathing condition (NB vs. HB) between groups (SR vs. FR) on SaO_2_ and HR. Sidak Holm *post-hoc* tests was used for multiple pair wise comparisons. Eta squared for main effects were calculated for the ANOVA, where the values of the effect sizes were considered as follows: 0.2 (small), 0.5 (medium), and 0.7 (large; Cohen, 1988). The statistical analyses were conducted at a 95% confidence level with the level of significance set at *p* < 0.05.

## Results

### SaO_2_ Recovery After Surfacing

**Figure 3:**
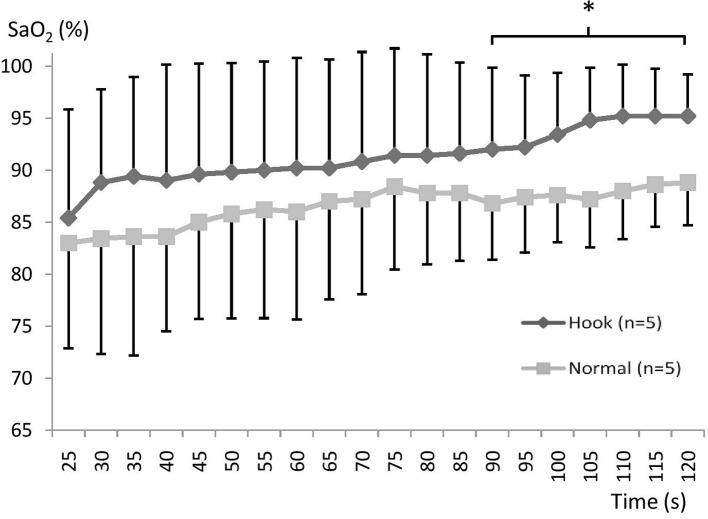
Mean (SD) SaO_2_ during recovery using normal or hook breathing after a 30 m dive in free immersion (FIM) for the group with slow recovery. * indicates *p* < 0.05 for the marked period.

**Figure 4:**
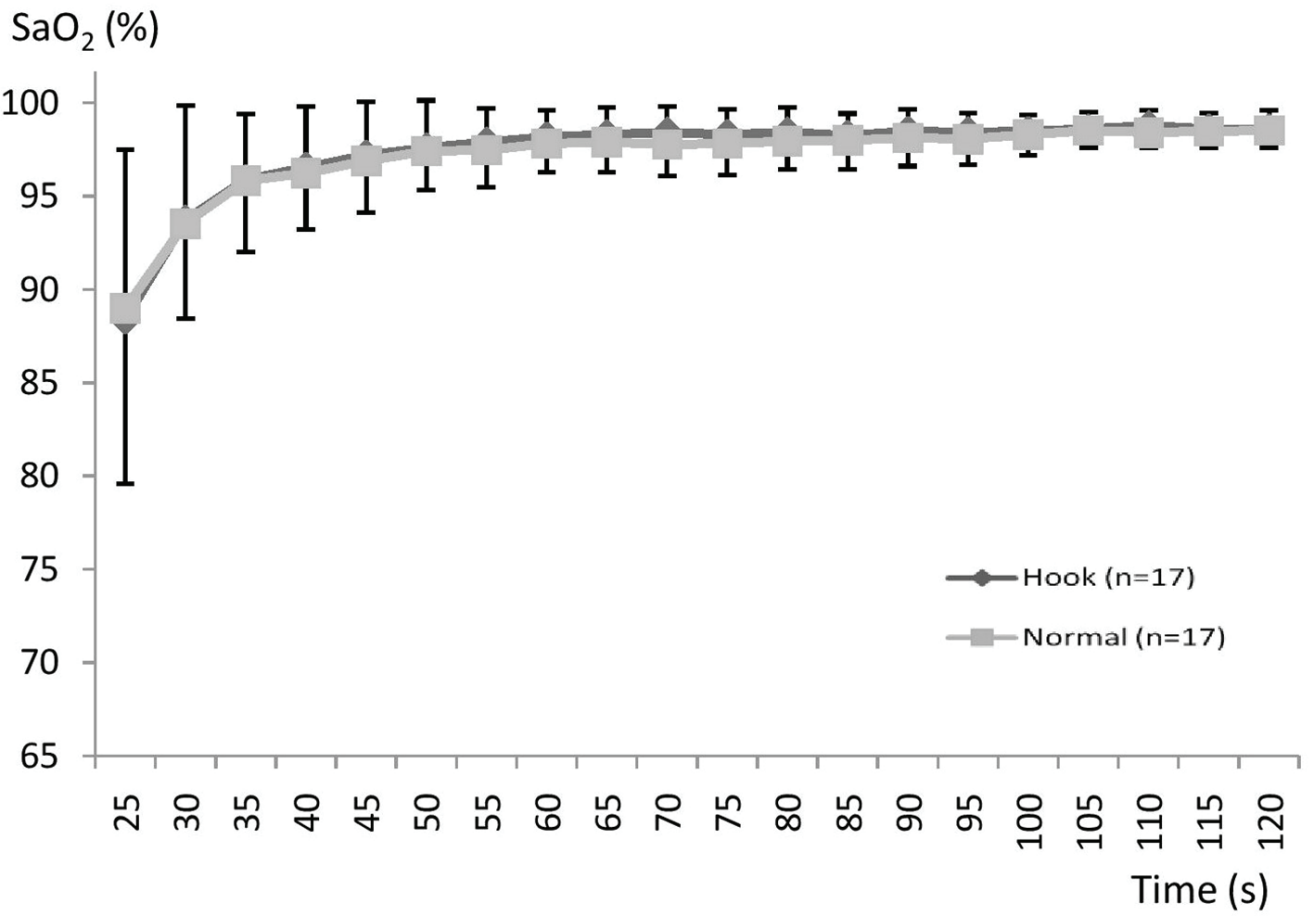
Mean (SD) SaO_2_ during recovery using normal or hook breathing after a 30 m dive in free immersion (FIM) for subjects with fast recovery.

The SR group responded with a more rapid SaO_2_ recovery during HB (Figure 3), while FR subjects showed no effect of breathing maneuvers (Figure 4). After surfacing SaO_2_ was higher in SR during the period 90 s (*p* = 0.045; $\eta_{p}^{2}$ = 0.09) to 120 s (*p* = 0.001; $\eta_{p}^{2}$ = 0.52) after surfacing SaO_2_ was significantly higher with HB (Figure 3). In SR subjects, the average time needed to reach 95% SaO_2_ was 60 s with HB and >120 s with NB (*p* < 0.05). The FR group did not show any differences in SaO_2_ between HB and NB at any point, and they recovered fully within 2 min in both situations (NS; Figure 4).

### Heart Rate Recovery After Surfacing

**Figure 5:**
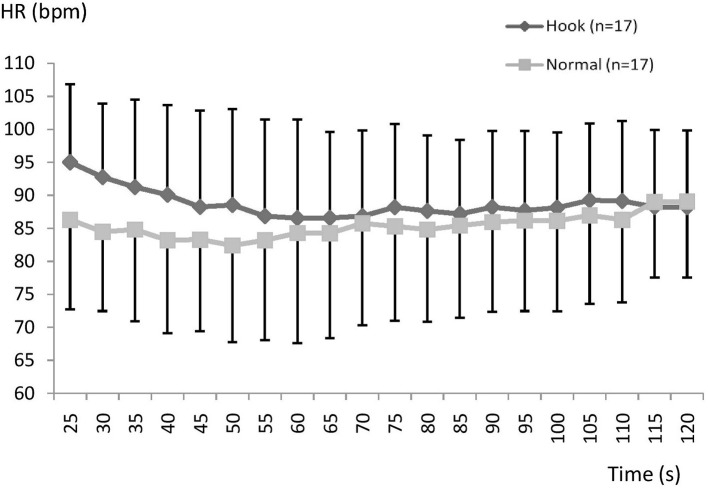
Mean (SD) heart rate during recovery using normal or hook breathing after a 30 m dive in free immersion (FIM) for subjects with fast recovery.

**Figure 6:**
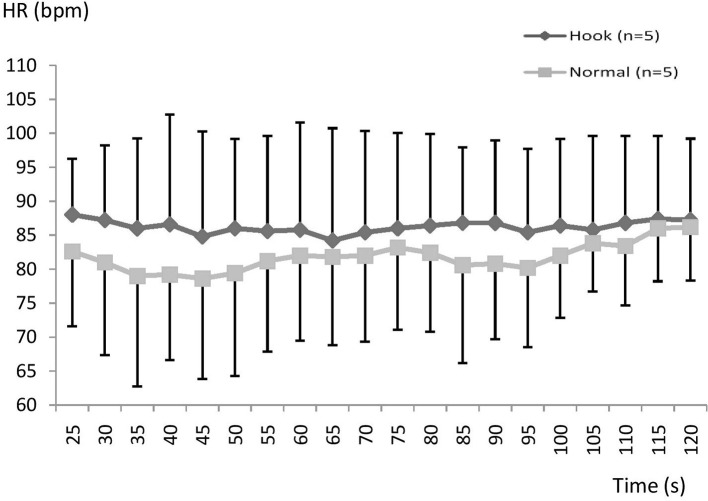
Mean (SD) heart rate during recovery using normal or hook breathing after a 30 m dive in free immersion (FIM) for the group with slow recovery.

There is a tendency throughout the recovery period for a higher HR during HB, especially early in the SR (Figure 5) but no significant difference found in the SR or FR group (NS; Figures 5, 6).

## Discussion

This is the first study examining the physiological effects of HB, a breathing pattern often used by freedivers and considered to counteract BO. The faster SaO_2_ recovery using HB in the group of subjects with delayed recovery is a novel finding. This suggests that the breathing method developed by the divers has a positive function in counteracting delayed recovery in our subject group likely suffering from mild pulmonary edema, in which the method is somehow increasing pulmonary gas exchange and thereby reducing the risk of BO.

In FR subjects, with apparently less proneness to pulmonary edema, the effects of HB were marginal, and it is suggested that their recovery is efficient enough with NB, at least after submaximal dives. As no direct diagnostic methods of pulmonary edema were available at this field setting, we base this interpretation on the correlation between recordings of SaO_2_ and lung comets revealing the presence of lung water in our methods development test. Other undetected factors could contribute to the differences in recovery rate between groups.

There could be several mechanisms responsible for the increase in pulmonary gas exchange associated with HB. We speculate that one major cause is the opening up of atelectatic areas of the lung with the increased intrapulmonary pressure and forcing out any fluid associated with pulmonary edema. Possible other effects of HB could be due to the increased alveolar partial pressure of O_2_ achieved, the extension of the respiratory membrane with increased intrapulmonary pressure increasing the surface area available for gas exchange, and also making the respiratory membrane thinner. We further suggest that other possibly contributing effects of HB could be related to changes in pulmonary ventilation/perfusion, venous return and blood pressure, or the increases in blood pressure achieved with HB, avoiding the drop in blood pressure just after surfacing. For determination of which mechanisms are responsible for the described effects, the breathing maneuvers should be further studied in the laboratory, and the effects should be studied in a larger number of divers in the sea at various conditions and include the first 20 s after diving, as the sample in our study was small and we could not record the initial phase of recovery.

The prevalence of divers with delayed recovery (5 of 22 divers) found in this study using submaximal dives, was much lower than in the study by Cialoni et al. (2012) where about 70% of the divers with the personal best around 57 m as our subjects had experienced symptoms. This suggests that the submaximal depth used in our study may not cause pulmonary edema in all divers with proneness. Diving depth beyond 40 m is identified as a risk factor for squeeze (Linér and Andersson, 2008) but our study for safety reasons focused on dives to a more moderate depth. Thus, the portion of divers in the studied category which could benefit from using HB after deeper dives may in fact be greater than the 23% identified in our study. Differences in recovery rate between equally skilled subjects after dives to a submaximal depth suggests that there are many factors affecting the proneness to pulmonary edema, which may not be directly related to the hydrostatic pressure, e.g., genetic factors (Cialoni et al., 2015) and training (Schagatay, 2011).

While in competition divers 30 m may be considered very moderate, in most other freedivers including spearfishers and food collectors, 30 m may represent the maximum reached. Our results show, as do earlier studies (Boussuges et al., 1999; Cialoni et al., 2012) that squeeze may occur also at this depth and our study suggests that the use of recovery HB could be motivated also in other groups of freedivers.

Further studies are required to reveal the effects of HB on recovery after maximal dives, but in competition, nearly all divers prefer to use HB as they consider it safer. It would also be interesting to register history of previous edema and measure lung volumes—especially the TLC/RV ratio, in order to better understand the causes of pulmonary edema in deep diving.

The slightly elevated HR during the recovery period probably reflects a higher physical exertion during HB than NB. That is, during HB, there is a greater respiratory muscle recruitment, requiring a higher energy demand, and also an increased venous return (Whinnery and Murray, 1990) would likely cause tachycardia *via* baroreceptors due to the Bainbridge reflex (Hakumäki, 1987).

We used pulse oximetry to identify rate of SaO_2_ recovery, to our knowledge, the only available method that is rapid enough to reflect the first minute of recovery after diving, where the diver is at most risk for BO. SaO_2_ values obtained by pulse oximetry have been found to correlate to symptoms from the lungs (Schagatay et al., 2015), and reported to be related to the presence of fluid in the lungs as suggested by spirometry (Linér and Andersson, 2008) and by pulmonary ultrasound (the present study). However, this method could not record the initial 20 s after surfacing, and underwater monitors would likely provide more complete data for the initial period. The delay of the start of measurements is caused by the time taken to reach the boat side, the hand dried, and finger blood flow fully established, but as long as there are no underwater monitors of SaO_2_, we believe this is an acceptable method to use. With the use of finger pulse oximetry, there is also a delay of the nadir saturation compared to more central measurements, which could be negative when compared to clinical methods, but with NB and HB, these effects should be similar, we do not see this would compromise our results. In fact the delay may be beneficial in this situation, as the value we monitor at 25 s may represent events occurring at a point closer to the dive termination.

Our results suggest that HB is highly efficient in speeding up recovery in individuals showing delayed SaO_2_ recovery most likely related to mild pulmonary edema, while in subjects without delay, the effect is insignificant. In dives closer to individual maximum depths, the beneficial effect may be expressed in a larger group of divers. This study suggests that the intuitive use of HB among freedivers is beneficial and that HB should be encouraged as a means to avoid BO after surfacing from deep dives.

## Data Availability

The raw data supporting the conclusions of this manuscript will be made available by the authors, without undue reservation, to any qualified researcher.

## Ethics Statement

Local Human Research Ethics Committee (Universidad Autónoma de Madrid) (CEI-70-1,257).

## Author Contributions

ES presented the original idea, after input from Lotta Ericsson in 2009. Pilot tests were done by ES and methods development by ES in 2016. FF and LR-Z collected the data during 2017. Analysis was performed by all authors and writing mainly by FF and ES.

### Conflict of Interest Statement

The authors declare that the research was conducted in the absence of any commercial or financial relationships that could be construed as a potential conflict of interest.

## Supplementary Material

SUPPLEMENTARY TABLE S1 Division of groups according to their SaO_2_ recovery rates. Subjects that did not always reach 95% SaO_2_ within 2 min after surfacing were assigned to the “slow recovery” group. Recovery data are shown in seconds (s).
