# Supplementary material for: Hook Breathing Facilitates SaO2 Recovery After Deep Dives in Freedivers With Slow Recovery
Source: Front Physiol. 2019 Aug 30;10:1076. doi: 10.3389/fphys.2019.01076 (PMC6729099; doi:10.3389/fphys.2019.01076)
Supplement: SUPPLEMENTARY TABLE S1 — Division of groups according to their SaO2 recovery rates. Subjects that did not always reach 95% SaO2 within 2 min after surfacing were assigned to the “slow recovery” group. Recovery data are shown in seconds (s). [file Table_1.docx]

| Fast (n=17) | 1 | 2 | 3 | 4 | 5 | 6 | 7 | 8 | 9 | 10 | 11 | 12 | 13 | 14 | 15 | 16 | 17 |
| --- | --- | --- | --- | --- | --- | --- | --- | --- | --- | --- | --- | --- | --- | --- | --- | --- | --- |
| HOOK (s) | 25 | 30 | 45 | 30 | 35 | 30 | 45 | 25 | 25 | 35 | 35 | 40 | 60 | 25 | 30 | 30 | 30 |
| NORMAL (s) | 25 | 25 | 35 | 25 | 50 | 25 | 35 | 30 | 30 | 35 | 45 | 40 | 25 | 35 | 35 | 35 | 60 |
| Slow (n=5) | 1 | 2 | 3 | 4 | 5 |  |  |  |  |  |  |  |  |  |  |  |  |
| HOOK (s) | 40 | 40 | 70 | 75 | 120 |  |  |  |  |  |  |  |  |  |  |  |  |
| NORMAL (s) | >120 | >120 | >120 | >120 | >120 |  |  |  |  |  |  |  |  |  |  |  |  |
